# Surgical Clinic, Memphis, Tennessee

**Published:** 1890-01

**Authors:** W. B. Rogers

**Affiliations:** Professor Principles and Practice of Surgery and Clinical Surgery


### HYDROCELE—STRICTURE OF URETHRA WITH PERINEAL FISTULA—PHIMOSIS—ORCHITIS—ANEURISM OF SUBCLAVIAN ARTERY—COLLES' FRACTURE—FRACTURE OF TIBIA.

BY W. B. ROGERS, M. D.,
Professor Principles and Practice of Surgery and Clinical Surgery.

[Surgical Clincal at Memphis Hospital Medical College, Month of November, 1889. Reported by S. H. Howard.]

CASE I. HYDROCELE.—Treated by carbolic acid injection.

Edward White, twenty-four years, resident of Memphis, suffered three years ago with gonorrheal inflammation of the testis, which on subsiding, left the organ apparently twice its normal size. His general health has been perfect, and he experiences only slight pain in the enlarged testis. Patient was placed supinely on the table, and the "tumor within the scrotum" examined. It was found confined to the testis—the cord not being involved—nor did it disappear on being elevated, hence in the differential diagnosis *varicocele*, *hernia*, *encysted hydrocele of the cord* and *congenital hydrocele* were all eliminated. Palpation determined the presence of fluid, thus excluding the various solid growths of the testes, and thereby reducing it to a question between hæmatocele and hydrocele. By means of a lighted taper, translucency was proven, and a positive diagnosis of hydrocele of the tunica vaginalis was made. The quantity of fluid was small, probably not exceeding three drams. By means of a hypodermic syringe a half dram was drawn off, clear and straw-colored. The hypodermic syringe was then filled with carbolic aid, and fifteen minims were then thrown into the sac, already containing about two and a half drams of fluid. No pain was experienced by the patient, who was instructed to report in ten days.

CASE II. HYDROCELE.—Carbolic acid injected.

Jas. Elbert, twenty-eight years, male, white, was brought to clinic by Dr. Etheridge, of Alabama. Patient had had "mumps" eight months previous, metastasis had taken place and there still remained some enlargement of the left testis. He was placed on the table, and ten of the class called to examine the "tumor within the scrotum." In shape and size it resembled a small pear. First by exclusion and then by test for translucency it was diagnosed hydrocele. The radical operation of incision was described; but the patient desiring to return to his home, it was decided to inject the sac with carbolic acid. The needle of hypodermic syringe filled with carbolic acid was first introduced, then with a small trocar, all the fluid (four ounces) was drawn off, and the carbolic acid was at once thrown in. Slight pain only was complained of by the patient. He went to his home in Alabama on the second day after the operation.

CASE III. ORCHITIS.

Robt. L., thirty-eight years, white, presented a "tumor within the scrotum" confined to the right testis, which was exceedingly sensitive to the touch. The enlargement was confined to the organ, yet the vas deferens was found very tender on the slightest pressure. The enlargement was of only four days duration, came without traumatic injury and was traceable to urethritis, the remains of which still were present. The case was one of acute (specific) orchitis and epididymitis. It was explained that the epididymitis of necessity preceded the orchitis, that both arose by reason of spread of the inflammation along the vas deferens. The following course of treatment was ordered: Morphia, hypodermically for reliet of pain, absolute rest in the bed, with elevation of the parts, and application of hot poultices. Thorough evacuation of the bowels by the use of calomel in full doses. It was urged that in any case of this kind, it would be found that the liver was torpid and the portal circulation very sluggish; that constipation usually was marked; that in some cases diarrhœa existed, but if so, it was the type of diarrhœa that corrected itself as soon as the liver was acted on by calomel. And that positive improvement begins at once after the calomel acts.

CASE IV. PHIMOSIS.—Circumcision.

Albert Grey, thirty-four, colored, presented very much contracted preputial orifice, with slight purulent discharge, apparently from non-specific balanitis. Had been unable to retract prepuce for some months, and unable to thoroughly cleanse and medicate through the existing small opening. Chloroform was administered, and the operation of circumcision performed—mucous membrane stitched to integument with continuous catgut suture.

CASE V. PERINEAL FISTULA.

Henry Green, twenty-two years, colored, was presented with perineal fistula through which most of the urine escaped at each evacuation of the bladder. He gave history of having received an injury in the perineum one year previous which was followed by marked swelling and induration, all of which gradually disappeared, leaving behind a stricture of the urethra as evidenced by difficult micturition, and very small stream. Four weeks ago the swelling returned in the perineum, became very painful and resulted in an abscess which opened spontaneously ten days before case entered hospital. The urethra was cocainized, and a twenty-seven bulbous sound passed readily as far as membranous portion of the urethra and was there arrested. Smaller instruments were used without success; until finally a filiform whalebone guide was passed and over this a dilator was with care passed to the bladder, the finger per rectum confining the location of the instrument. The stricture was rapidly dilated and instrument withdrawn. The hemorrhage was slight. A No. 19 American steel sound was then passed into the bladder, after which the patient easily urinated a full stream, *per vias naturales*, no water coming through the fistula.

Patient was presented at each clinic during the month, and Nos. 17, 18, 19 sounds were easily passed; parts much improved, very little water passing the fistulous opening.

CASE VI. ANEURISM OF LEFT SUBCLAVIAN.

John Bryan, male, white, thirty-four years of age, was presented. Six weeks previously he, while in a difficulty received two stab wounds just below the clavicle in left side and just internal to coracoid process. Both wounds ranged in the direction of the third portion of subclavian artery. The wounds healed kindly. At present examination he presents slight tumor just below and apparently coming from beneath the clavicle, just over or upon the subclavian or junction of that artery with the axillary. Pulsation is distinct. The bruit is readily perceived. Slight pressure dispels the tumor with its rasping murmur. The diagnosis was plain. An aneurism of traumatic origin. Treatment advised was pressure by tourniquet. If this fails then the ligature to third portion of artery if tumor grows.

CASE VII. COLLE'S FRACTURE.

James K., male, twelve years of age, white, fell a distance of six feet, most of his weight being received on the palm of the left hand. Examination revealed fracture of the left radius, just above the articulation with the carpal bones—slight displacement backward of lower fragment. Fracture was reduced and Levis' perforated splint applied.

CASE VIII. FRACTURE OF LEG.—Treated with plaster Paris.

Henry W., male, white, ten years of age fell and produced a fracture of the bones of the leg. He was presented six days after the injury was received. Examination detected motion at junction of middle with lower third of leg. There was scarcely any deformity. No effort was made to secure crepitus, because of the evidence offered by the undue mobility. The swelling was not very marked, and was on the decline. The limb was enveloped in cotton wadding, and plaster of Paris applied over the wadding. The plaster set promptly, and the boy was carried home to be returned at future clinics.

